# A systematic assessment of stress insomnia as the high-risk factor for cervical cancer and interplay of cervicovaginal microbiome

**DOI:** 10.3389/fcimb.2022.1042663

**Published:** 2022-12-06

**Authors:** Ravina Rai, Manisha Nahar, Deepali Jat, Neelima Gupta, Siddhartha Kumar Mishra

**Affiliations:** ^1^ Department of Zoology, School of Biological Sciences, Dr. Harisingh Gour Central University, Sagar, India; ^2^ Vice-Chancellor, Dr. Harisinsgh Gour Central University, Sagar, India; ^3^ Department of Biochemistry, University of Lucknow, Lucknow, India

**Keywords:** cervical cancer, cervicovaginal microbiome, physiological stress, insomnia, cytokine profile, inflammation, oxidative stress

## Abstract

Cervical cancer is a dreaded form of cancer in women, the fourth most common cancer, with around 0.3 million females suffering from this disease worldwide. Over the past several decades, global researches have focused on the mitigation of cervical lesions and cancers and have explored the impact of physiological and psychological stress and insomnia on cervical pathogenesis. Furthermore, disruption of the cervicovaginal microbiome profiles is identified as an added high-risk factor for the occurrence of cervical cancer. The physiological regulation of stress has an underlying mechanism controlled *via* hypothalamic pituitary adrenal (HPA) and sympatho-adrenal medullary (SAM) axes. Disruptions in these axes have been identified as the factors responsible for maintaining the homeostasis balance. Recent studies on microbiomes have offered novel ways to combat cervical cancer and cervix infection by exploring the interplay of the cervicovaginal microbiome. Moreover, the integration of various immune cells and microbiome diversity is known to act as an effective strategy to decipher the cervix biological activity. Cytokine profiling and the related immune competence, and physiological stress and insomnia impart to the regulatory networks underlying the mechanism which may be helpful in designing mitigation strategies. This review addressed the current progress in the research on cervical cancer, HPV infection, immune cell interaction, and physiological stress and insomnia with the cervicovaginal microbiome to decipher the disease occurrence and therapeutic management.

## Highlights

• Cervical Cancer and Cervix Lesion: Abnormalities in squamous and glandular cells cause cervix lesions and promote cervical cancer.
•Diverse Profile of the Cervicovaginal Microbiome: Some species of *Lactobacillus* such as *L. crispatus, L. gasseri* and *L. jensenii* produce lactic acid and hydrogen peroxide, which inhibit the growth of harmful bacterial and viral pathogens.
•Physiological Stress: Serves as life-threatening situations of which emotional stress, prolonged starvation, and physical trauma may highly interrupt homeostasis.
•Stress Insomnia: Insomnia is usually a result of stress that can keep your mind active at night, making it difficult to sleep.
•Cytokines and Chemokines Profiling: Cytokines and chemokines activities are the major players in fighting against antigen and pathogen infections by coordinating and activating the adaptive immune response.
•This review has explored the impact of stress on cervix physiology and elaborated on the extent of insomnia as the risk factor for cervical cancer and its association with the alterations in the cervicovaginal microbiome, especially in working and stressed women.
•Compiled the profile of cytokines and chemokines and correlated with the stage-specific conditions of cervical cancer during stress conditions and focused on the unique changes that characterize early-stage detection of cervical cancer.
•The recommendations drawn are important to prevent persistent and life-threatening cervical lesions; therefore, stress insomnia and unhealthy the cervicovaginal microbiota acting as severe causes of cervical cancer promotion must be treated at early onset.

## Introduction

Cervical cancer is one of the most common causes of cancer related deaths in women worldwide which is ranked fourth of all cancers with increased mortality to 0.3 million globally in 2020 (Sung et al., 2021). Repertoires indicate that 90% of all cervical cancer cases occur in countries with low- and middle-income mainly because of inadequate screening, lack of early detection systems, and lesser preventive measures for both pre-cancerous and cancer stages (Elemam and Bakhit, 2018). There are many factors responsible for cervical cancer such as smoking, alcohol consumption, destructive free-radical mediated oxidative stress, defects in the endogenous antioxidant defense systems and human papillomavirus (HPV) infection (Yang et al., 2022). Currently, over 100 different types of HPV are described, amongst which at least 14 types are cancer-causing or known as high-risk types for cancer development. Two of the HPV types, HPV16 and 18, are associated with 70% of cervical cancers and pre-cancerous lesions in the cervix (Xu et al., 2020). HPVs can infect basal epithelial cells or the inner lining of cutaneous tissues or mucosal tissues. Mucosal typed HPVs infect the anogenital epithelium or the cervical regions in women and thus are associated with cervical cancer and precursor lesions. HPVs are grouped as high-risk and low-risk types, amongst which the high-risk group HPVs are critically associated with cervical cancer whereas other HPV types are less frequently found in cancers (Burd, 2003). Infection with high-risk HPV types interferes with cellular functions with notable alterations in gene signatures. Several genes that are down-regulated are primarily involved in the regulation of cell growth and proliferation, and interferon (IFN)-responsive genes (Lukac et al., 2018). A high mortality rate was perceived in the aged women (over 65 years) due to the advanced stage of the disease, and that amongst them 50% of subjects were tobacco smokers and 50% were non-smokers (Zeng et al., 2012). We have shown that passive smoking acts as an independent and combined risk factor of cervical intraepithelial neoplasia 1 (CIN1) and that HPV viral load and alcohol consumption synergistically increased the risk of CIN1 among high-risk HPV-positive patients (Kim et al., 2012). Tobacco smoking has been considered as a well-established risk factor for cervical precancer and cancer and epidemiological studies have suggested their associations with the risk of cervical cancer. This implicated the role of smoking as a stressor in the etiology of cervical cancer in passive-smoking women in comparison to non-smoking women (Kim et al., 2012). Reports show that the cervicovaginal microbiome plays significant roles in the prevention of cervical infection. During cervix infection, if the HPV and other carcinogenic agents enter inside the women’s reproductive tract then the vaginal microbiomes have the capabilities to wash out and neutralize all the infecting pathogens in the process generally called as virus and carcinogen clearance (Mitra et al., 2016). Vaginal microbial diversity was shown to be associated with the severity of CIN disease and that microbes could contribute to regulating the persistent viral infections and cervical lesions progression. CIN1 progression was recognized for having the role of the cervical microbial community working synergically with HPV in cervical cancer (Wang et al., 2022). Over the age of 65 years, insufficient sleep, disturbance of the biological clock, and long-term stress conditions can cause stress insomnia in women. Stress insomnia can reduce the ability to fight off a common cervicovaginal infection and increase the risk of developing cervical cancer (Sims et al., 2021). Physiological stress and sleep disorder were shown to affect the level of cytokines and immune response, both produced and released during sleep and exerted direct effects on the microbiome (Garbarino et al., 2021). Evidences suggest that the cervicovaginal microbiome may impart actively in modifying the local host immune response (cytokines and chemokines) which has been observed in other sites of mucosal differentiation (Anahtar et al., 2015). The condition of stress generation and its level causes the secretion of a high number of inflammatory cytokines and chemokines which affects the composition and profile of microbiota and imparts to cervical infections (Xiao et al., 2011). According to recent advanced studies, women who are working frequently late at night and travel excessively may have different cytokine profiles as compared to normal ones. The cytokine profile included higher levels of antiviral interferons (IFN-α2 and IFN-γ), regulatory interleukins (IL-4, IL-5, IL-13 and IL-10), and pro-inflammatory cytokines and interleukins (IL-1α, IL-1β, IL-8, IL-6, IL-12, TNFα and MIP-1α) (Moscicki et al., 2020).

**Figure 1:**
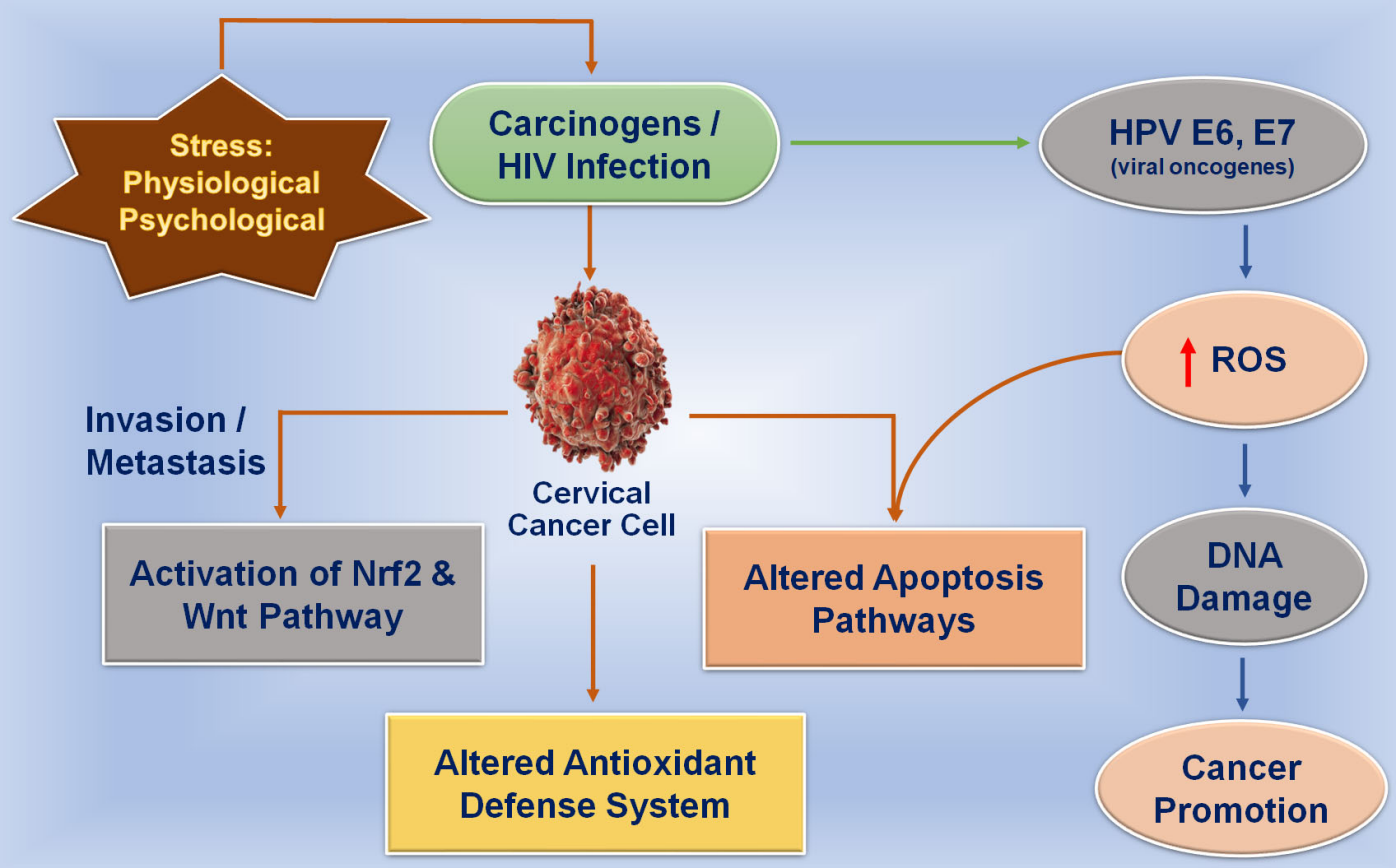
Effect of physiological and psychological stress on oxidative stress and viral infection in the cervix region. Physiological and psychological stress directly affects the generation of oxidative stress in tissues that are correlated with the initiation of DNA damage in the cervical epithelium which further promotes tumor development.

Taking the impressions from earlier reports, some gaps in the current state of knowledge were identified which mainly included the elaboration of the complete taxonomic and functional profiling of the cervicovaginal microbiota. As the whole genome and 16S rRNA gene amplicon sequencing may have false-positive results and low specificity due to contamination, it may cause variable detection in the case of cervical cancer (Nieves-Ramírez et al., 2021). Another major gap in the current state of knowledge is how the cervicovaginal microbiota interacts with host cells, in terms of local immune mediators, during the pathogenesis of pregnancy related complications and stage-specific conditions of cervical cancer. The incidence of gynecological malignancies, especially cervical cancer, has increased during pregnancy which is largely attributable to older age pregnancies (Beharee et al., 2019). It was reported that both pregnancy and HPV infection caused an increase in the vaginal bacterial microbial diversity and richness and that the bacterial composition was also influenced by pregnancy (Chen et al., 2019). In this review, we have explored the impact of stress on cervix physiology and elaboration of insomnia as the risk factor for cervical cancer and alterations in the cervicovaginal microbiome, especially in working women. The effect of physiological stress on oxidative stress and viral infection in the cervix region was shown by interacting aspects of the physiological and psychological stressors to the generation of oxidative stress which is further correlated with the initiation of DNA damage in cervical epithelium which causes promotion of cancer (**Figure 1**). The review aimed to create an account of the currently available literature on stress insomnia associated with cervical cancer and its effects on the cervicovaginal microbiome. We have discussed the complete profiling of cytokines correlated with stage-specific conditions of cervical cancer during stress conditions in women and focused on the unique changes that characterize early-stage detection of cervical cancer.

## Physiological stress and the molecular mechanism

**Figure 2:**
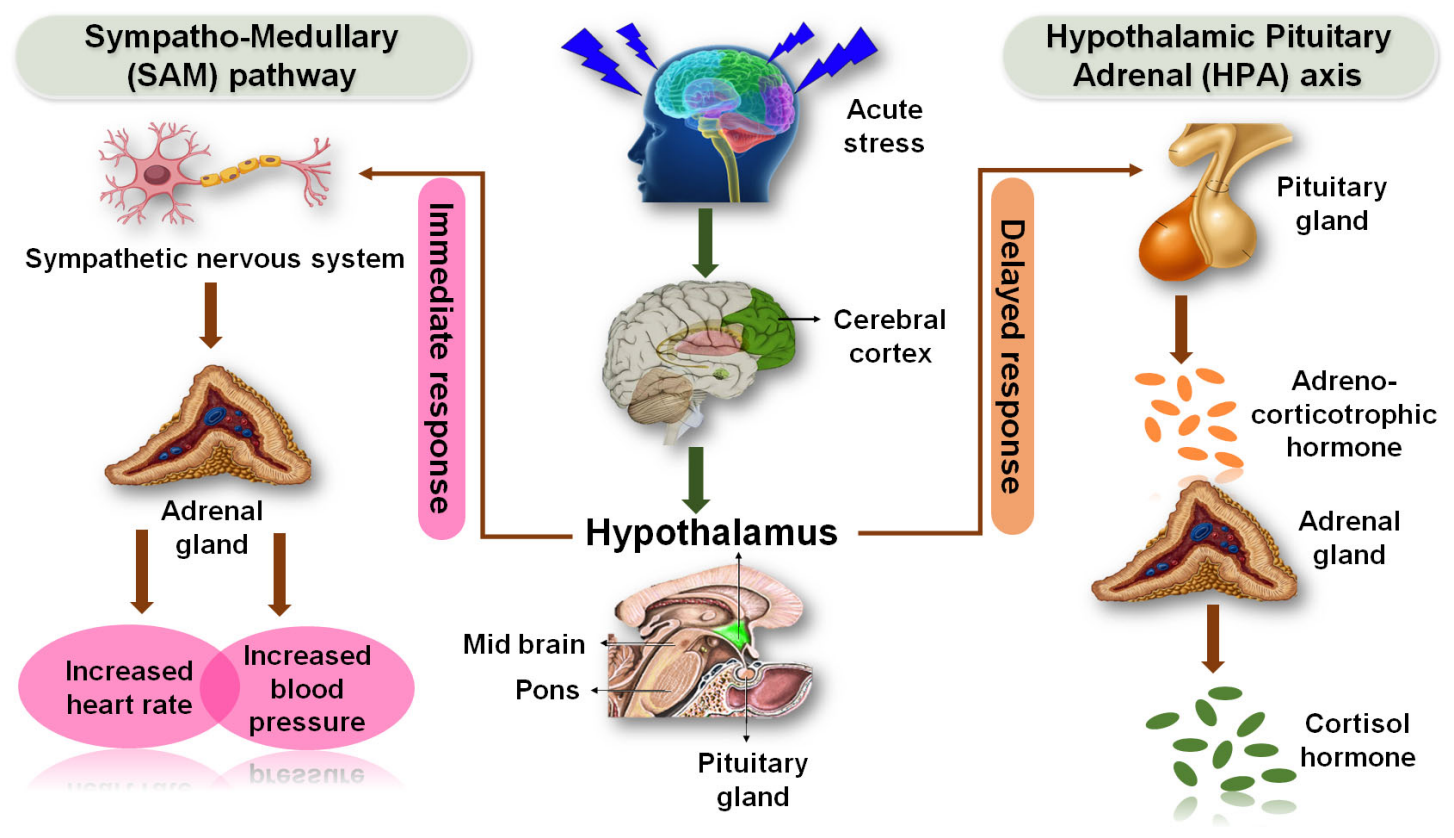
Stress responsive pathways of nervous and endocrine systems. Effects of acute stress on the nervous system and endocrine system that are usually mediated through SAM and HPA axis pathways.

Physiological and biological stresses of an organism create the responses towards a stressor like an environmental condition (Ash et al., 2021). Stress is the physiological mechanism of the body that has a response system to conditions like threats, challenges, and psychological barriers. Physical, mechanical, and mental stimuli caused several changes in organisms’ environment which are reflected by multiple systems in the organism body (Huzard et al., 2021). Environmental toxicants have drastically impacted on physiology and have led to carcinogenic developments (Ishfaq et al., 2021; Ishfaq et al., 2022). The stress responsive pathways of nervous and endocrine systems are depicted in **Figure 2** which comprehensively demonstrates the effects of acute stress on these physiologies and the involvement of the sympathoadrenal medullary (SAM) and hypothalamic-pituitary-adrenal (HPA) axis pathways. In humans and most mammals, the endocrine system consisting of the HPA axis and the autonomous nervous system, are the two physiological systems responding to stresses as frontier physiological mechanisms. Another physiological system, SAM axis responds through the sympathetic nervous system and may activate during the flight-or-fight condition, while the parasympathetic nervous system maintains the homeostasis of body (Zhang et al., 2019). The HPA axis, the second major physiological stress-response center, regulates the release of cortisol from adrenal gland which upon release regulates different functions of body such as immunological, metabolic, and psychological processes. Both stress response systems, the HPA and SAM axis, are regulated by several regions of the brain mainly the hypothalamus, prefrontal cortex, limbic system, stria terminalis and amygdale (Everly and Lating, 2019). The effects of the various psychological stressors are linked to different immune responses such as natural and specific immunity. Natural immunity produces swift response through immune cells that could attack many pathogens and cause inflammation. Their front-line fight in the body engages in efficient attacks on specific invaders *via* the T and B cells, which fight pathogens like viruses, and exert cellular and humoral responses (Morey et al., 2015). Studies have denoted that these mechanisms alter the brain memory functions, immune functions, and metabolic functions due to stress, and act as causes for some chronic diseases like cervical cancer. Severe or chronic stress has been associated as a common risk factor for several mental illnesses (Everly and Lating, 2019). There are five different types of stressors, classified as chronic stressors, acute time-limited stressors, stressful event sequences, brief naturalistic stressors, and distant stressors (Segerstrom and Miller, 2004). A chronic stressor is associated with long-term stress, an acute time-limited stressor is a short-term challenge, the stressful event is a stressor involved in regular stress condition, a brief natural stressor is an event that is normal not very challenging, and a distant stressor is a stressor that is not immediate (Russell and Lightman, 2019). Acute time-limited stressors last for less than two hours which regulate natural immunity and downregulate specific immunity. This type of stress causes increased expressions of granulocytes, natural killer (NK) cells, IgA. and IL-6, and increases the level of cell cytotoxicity. Succinct naturalistic stressors stimulate a shift from Th1 cellular to Th2 humoral immunity, while they decrease T-cell proliferation and NK cell cytotoxicity (Russell and Lightman, 2019). Stressful events influence immune response *via* the generation of free radicals; however, major observations are decreased T-cell growth and proliferation and increased NK cell cytotoxicity (Segerstrom and Miller, 2004). Cancer patients’ survival has a relationship with chronic stressors which perturbs the psychoneuroimmune axis and exerts profound Th2 immune response, especially characterized by inflammatory overactivation of CTLs, IFN-γ, and IL-5 (Reiche et al., 2004; Segerstrom and Miller, 2004). Chronic stress also influences the humoral immunity and causes shift toward Th2 immunity and causes a decrease in IL-2 and T-cell proliferation which are associated with decreased antibody response to some vaccines. However, distant stressors often do not elicit consistent changes in immune function (Eimonte et al., 2021).

**Figure 3:**
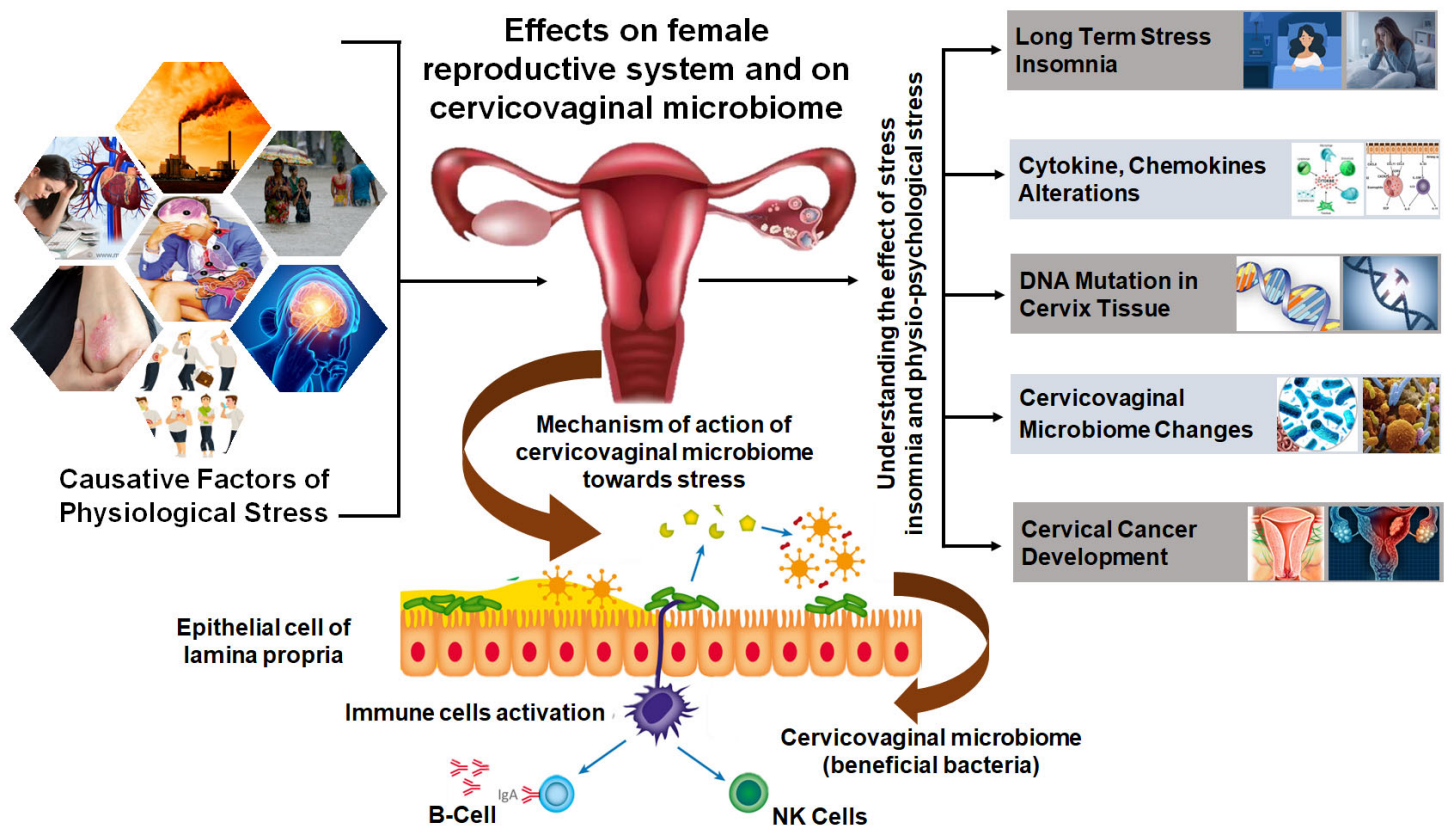
Comprehensive summary of stress responses and risk factors affecting cervical physiology and cancer development. Different causative factors impacting negative consequences of the physiological stress on female reproductive system which ultimately lead to modulating DNA integrity, inducing inflammatory cytokines and chemokines profile, and altering the cervicovaginal microbiota, and consequently causes the development of cervix lesions and cervical cancer.

Homeostasis is a central concept of stress in human biology with most of the biochemical processes maintaining their equilibrium stage also called steady state or homeostasis. External and internal environmental factors continually act and cause disruptions to the homeostasis. The physiological state of an organism moving around a homeostatic point maintains the optimal growth and development of the organism (Kotas and Medzhitov, 2015). Physiological stresses like emotional perturbances, prolonged starvation, and physical trauma can highly disrupt homeostasis and may create a life-threatening condition. Cervical and ovarian cancer are the two major gynecological malignancies of women which are drastically influenced by physiological stressors (Chen et al., 2021). A positive correlation between chronic stress and cervical cancer progression was drawn showed that long-term stress stimuli may activate the sympathetic nervous system and the HPA. This led to the release of stress hormones, especially catecholamines and glucocorticoids, which act on β-adrenergic receptors, dopamine receptors, and glucocorticoid receptors. The communications between stress hormones and receptors could produce a series of physiological effects in the case of cervical cancer (Huang et al., 2016; Chen et al., 2017). The catecholamine neurotransmitter dopamine regulates various physiological functions of the central nervous system which was shown to promote the maturation and normalization of ovarian cancer (Moreno-Smith et al., 2013). Another type of stress responsive hormone glucocorticoids is widely used in clinics as anti-inflammatory and immunosuppressive agent. Glucocorticoids were shown to promote tumor cell survival, metastasis, and drug resistance in ovarian cancer. It could also affect the life cycle of HPV, interfered with the p53 activity, and reduced the persistence of HPV infection and resistance to cervical cancer patients (Shi et al., 2012; Karvonen et al., 2020). Continuous HPV infection remains to be the main reason for the occurrence and development of cervical cancer and that the risk of cervical cancer may increase multi-fold by addition of severe stress such as bereavement. Chronic stress caused neuroendocrine disorders that led to changes in the biological behavior of tumor cells which links psychological stress as the key player in cervical cancer progression (Kennedy et al., 2014). Therefore, it is advised that regardless of the cause of cervical cancer and its clinical condition, physiological and psychological stress needs to be regulated as an important part of its prevention or treatment. A comprehensive summary of stress responses and risk factors affecting cervical physiology and cancer development is presented in **Figure 3** which mainly demonstrates different causative factors impacting the negative consequences of physiological stress on the female reproductive system.

## Relationship between stress and cytokine profiles in cervical cancer

The relationship between stress and immunity with cervical cancer under observations of behavioral and environmental variable has been aimed to establish. Patients diagnosed with cancers have shown several stress and anxiety-related psychiatric and physiological disorders as risk factors that included anxiety, depression and distress reactions, and adjustment disorders as the major stressors (Lu et al., 2019). The psychological intervention of physiological and emotional stress was associated with the improvements in the quality of life and adaptive immunity, and survivorship of cervical cancer patients (Nelson et al., 2008). Psychological factors have been shown to play important role in the progression of different types of cancers with a notable number of stressed cases in cervical cancer patients with unclear psychologic distress at the time of diagnosis. Cervical cancer patients under the influence of stress disorders or a stressful life event were at a higher risk of mortality as compared to cancer patients seeking treatment of stress. Women diagnosed with cervical cancer have shown a history of psychological and oxidative stress that has been associated with a higher risk of mortality (Lu et al., 2019). The study examining the potential influence of stress on the cervical cancer-specific mortality on a large number of patients population showed a higher-risk to those patients who had been clinically diagnosed with any of the three kinds of psychiatric disorders; depression, anxiety, and stress-reaction and adjustment disorders (Lu et al., 2019). Emerging evidence from experimental and epidemiologic studies have demonstrated that psychological anxiety and distress can affect and modulate the progression of many cancer types (Mcbride et al., 2021). Physiological stress instances may cause increased cell proliferation, growth, tumorigenesis, angiogenesis, and tumor invasion *via* activation of the adrenergic pathway. However, chronic stresses may cause reduced cellular innate immunity response through both the adrenergic and glucocorticoid receptors on immune cells (Yeh, 2021). These pathways and their mechanism of action may play an important role in solid malignancies such as cervical cancer in women. Certain studies have provided information on whether psychological anxiety and distress are associated with the initiation of cervical cancer progression, diagnosis, and prognosis (Gupta et al., 2019). Stressful life events including divorce and bereavement may adversely affect cervical cancer survival yet the regulatory systems and controlling prognostic factors needed elaboration. It has been estimated that stressful social events such as divorce or widowhood may be associated with a delayed cancer diagnosis, affects prognosis, and lead to limited and costly treatments among patients with cervical cancer (Kohli and Dalal, 2020). We have illustrated in this review that patients diagnosed with cervical cancer have shown highly increased levels of several stress-related psychiatric disorders such as anxiety, depression, and stress-related reactions.

Cervical cancer occurrence is shown to be higher during an early age as the stress level rises suddenly which causes a resurgence of its peak leading to comorbid conditions and erroneous screening and diagnosis (Cvitanović et al., 2020). Cervical cancer patients showed the prevalence of depression and anxiety at about 52 and 66% respectively (Yang et al., 2014). Patients with cervical cancer stage II at the period of 4-6 months of diagnosis showed significantly high anxiety scores. Hope, optimism, and general self-efficacy accounted for about 30-35% variance of depression and anxiety in a hierarchical regression analysis which was identified as predictors of distress in cervical cancer patients (Yang et al., 2014). The prevalence of anxiety and depression was in about 50% of cancer patients as compared with those without cancers. Whereas obscure and untreated anxiety and depression were correlated to cause difficulty with symptoms’ control, poor prognosis, treatment failures, and prolonged recovery time towards the impaired quality of life (Yang et al., 2013). Cervical cancer patients are uniquely characterized by aggravated psychological distresses in addition to general distress which further adversely impacts on to cancer diagnosis and treatment. The score of anxiety was at a poor level due to emotional distress amongst patients with gynecological cancer, especially cervical cancer which showed a higher prevalence of depression (7-11%) and anxiety (53-62%) (Ferrandina et al., 2012). Distress-anxiety related disorders or stressful life events of patients are thus likely to impact disease occurrence, diagnosis and prognosis, and therapy. The increase in stress was associated with cancer diagnosis with a mixed effect on diagnostics and treatment as well as the status of living life with cancer and after therapy (Zhang et al., 2020). Acute stress gives a positive feedback for HPV and also promotes the transformation of the cell, wherein HPV caused disrupted cell signaling in the epithelial cells of the cervix and induced transformation into cervical cancer mainly by promoting the two pathways associated with metastasis and apoptosis (Curty et al., 2020). HPV infection being an important carcinogenic factor related to the incidence of cervical cancer has also shown exaggerated outcomes when associations with sexual activity, early first and premature sexual activity, sexually transmitted diseases, premature and disturbed episodes of pregnancy and fertility, excessive use of oral contraceptives and immunosuppressants, and smoking and alcohol (Shannon et al., 2017). A cervical screening study showed that high school and below, early fist sexual life (≤ 19 years), more than one sexual partner, and use of oral contraceptives were risk factors of HPV infection (Yang et al., 2022).

Physiological and psychological stresses stimulate the body’s responses to cause the release of catecholamines and glucocorticoids which exert cytokines balancing response upon interacting with corresponding receptors on immune cells. These responses are variably dependent on the nature of stressors and the mechanism of responsiveness that mainly includes production of cytokines as the mediators of inflammation. Cytokines are the low molecular weight proteins that mediate cell-to-cell communication. They are synthesized by immune and stromal cells, mainly endothelial cells and fibroblasts, and then they regulate cell growth, proliferation, differentiation, cell survival and death, immune cell activation, and cell migration (Showalter et al., 2017). Chronic stressors critically impact on the cytokines profile as compared with chronic stress hormonal signaling (Chen et al., 2021). In particular, the expression of IL-1, IL-2, IL-6, INF-γ, TNF-α, TGFβ, EGF, VEGF, and other growth factors are relatively more associated with chronic conditions as compared to other cytokines (Ho et al., 2021; Lu et al., 2021; Zhou et al., 2021). The reason behind this concept is that there are few cytokines, interleukins, and chemokines which have higher circulating levels as compared to other stress mediators, especially in asymptomatic individuals. Per se the circulating levels of IL-6 are usually higher than those of other cytokines in asymptomatic patients. While the level of cytokines and other immune responses vary from person to person because every individual has different immune response optimization against infectious particles (Lu et al., 2021). In assessing the hormonal aspect, the effect of the academic stress on the production of the Th1-cytokines (IL-2, IL-1β, IL-6, IL-8, IFN-γ, and TNF-α) and Th2-cytokines (IL-4, IL-1ra, IL-5, and IL-10) was analyzed on a group of medical or health professionals at the three stages; baseline stage (at the beginning), midterm, and final academic examination stage (Myint et al., 2021). The level of cortisol and cytokines in plasma during the three stages was measured which showed that their levels at the last two stages were comparatively significant as compared to that with the baseline (non-stress period). Observations showed that the level of stress induced during the final examination stage was highest in individuals with a significant increase in the release of cortisol. This caused a shift in Th1:Th2 cytokines balance towards Th2 with an increase in the levels of IL-1ra, IL-4, and IL-5. Whereas the midterm stage did not show any significant change in the levels of Th1-cytokines except for TNF-α and increased IFN-γ levels, yet these changes were further reduced in the third stage. Th2 cytokine (IL-1ra) showed a positive correlation with Th1 cytokines (IL-2 and IFN-γ) in the second stage and IL-6 in the third stage (Cohen et al., 2021). Though cortisol was positively correlated with the levels of IL-8 in the last stage, the heart rate showed a negative correlation with IL-10 in the first and last stages. Thus, exam stress was shown to downregulate the levels of Th1-cytokines with selective upregulation of Th2-cytokines. Conclusively, cortisol has been identified to play a key role in facilitating the release of Th1-mediated cellular immune response which led to an increase in the vulnerability towards infectious diseases including cervical lesions and cancer (Glaser et al., 1987). The study concisely presented highly relevant evidence towards the secretions of cytokines and chemokines during stress conditions.

During the state of chronic stress and inflammation, cytokines can also engage in inducing cell transformation and malignancy. At different stages of cervical cancer progression, it secretes cytokines and increases the level of secondary messenger, adapter protein, and receptor protein associated with cell proliferation and multiplication (Carrero et al., 2021). Studies have shown interesting observations during stress conditions which showed that if any infectious particle, pathogen, virus, or bacteria enters inside the cervical region the body prompts to induce both innate and adaptive immune response activities (Nelson et al., 2008; Nie et al., 2020; Xu et al., 2020; Yang et al., 2022). IFN-α and IFN-β are the main cytokines expressed downstream to cause activation of the innate immune system. As well as the cytokines associated with an adaptive immune system, mainly including IL-6, IL-8, IL-12, IFN-α2, TNF-α and MIP1α, are also associated with the inflammatory responses in the cervix infections (Zhou et al., 2021). These are produced upon activation of macrophages and dendritic cells, NK cells, and activated T cells. A diverse expression of inflammatory cytokines and chemokines has been observed, especially the expression of antiviral molecules (IL-12, IFN-γ, and IFN-α2), regulatory interleukins (Il-10, IL-13, IL-4, and IL-5), and pro-inflammatory molecule (TNF-α, MIP-1α, IL-1α, IL-1β, IL-6, and IL-8), as the immune-related predictive signature in the prognosis of cervical cancer patients (Nie et al., 2020).

**Figure 4:**
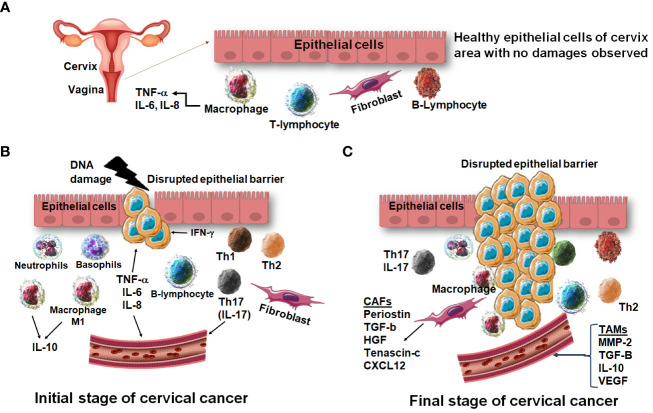
A comparative immunological profile of cervical epithelium. **(A)** The normal epithelium shows a balanced expression of cytokines and chemokines. **(B)** The early induction of cervical cancer shows DNA damage followed by overexpression of pro-inflammatory cytokines and chemokines. **(C)** At the advanced stage of cervical cancer, the cervical epithelium is disrupted, and migration of cells is observed.

The initial stage of cervical cancer marks a specific secretion of some amount of innate immunity related cytokines which help in clearance of viral and other pathogens. While the peak stage of cervical cancer marks the secretion of different kinds of inflammatory cytokines which are responsible for tissue damage and inflammation in epithelial cells of the cervix and vagina (Otter et al., 2019). Cytogenotoxicity has been associated with the nutritional genetic instabilities in cancer patients under different therapies especially in radiotherapy (Paz et al., 2019) and therapy has affected various biochemical markers especially inflammatory cytokines and chemokines in cancer patients (Paz et al., 2018). Thus, analyzing the comprehensive profile of inflammatory cytokines and chemokines relating to cervical cancer progression became an intrinsic module in cancer progression and therapeutic strategies as presented summarily in **Figure 4**.

## Cervicovaginal microbiome profiles and cervical cancer

There is a diverse microbial spectrum in the body complementing a microbiome that contains a complex mutualistic host-microbial relationship which play crucial roles in the development and physiological function of the body including immune system modulation and inflammation (Sims et al., 2021; Wu et al., 2021; Zhou et al., 2021). Cervical microbiota has identified a diversity of microbial species as possible modifiers of the cytokine profile of the cervical microenvironment, especially during increased stress conditions including physical, mental, and emotional stress (Curty et al., 2020). Numerous cervical cancer related studies have a focus on exploring the relationship between cervical microbiota and inflammation as the key drivers of cervical cancer (Nelson et al., 2008; Audirac-Chalifour et al., 2016; Shannon et al., 2017; Showalter et al., 2017; Torcia, 2019). The cervicovaginal microbiome contains unicellular organisms present in the cervix gut. They are classified into five different groups on the basis of 16S rRNA (30s subunit of the ribosome) high-throughput sequencing (16S-HTS) of the bacterial species (Kyrgiou et al., 2017). These different groups are commonly called as community state types (CSTs), the term firstly coined by Ravel (Ravel et al., 2011). CSTs are clustered into five groups, amongst which four were dominated by *Lactobacillus* species mainly *L. gasseri, L. crispatus, L. jenseniias*, and *L. iners* (Andralojc et al., 2021). Other commonly referred CST species are *Anaerobic cocci* and *Candida albicans*. Women with long-term stress conditions have been shown to develop symptoms like abnormal vaginal discharge, odor, and tissue damage in epithelial cells of the cervix area, and physiological and cellular inflammation, and have been diagnosed with bacterial vaginosis. However, both asymptomatic and symptomatic women are more likely to have HPV infection which drastically affects the cervicovaginal microbiota and other infections (Chorna et al., 2020). According to recent studies, vaginal microbial communities are usually dominated by one or a few having the capabilities to survive in a very low pH range, subsequently be able to actively participate, and become able to live on carbon sources derived from the host (Anahtar et al., 2015; Torcia, 2019; Chee et al., 2020; Castanheira et al., 2021). Amongst the likely sources are glycogen and mucin that are present in the vaginal region, yet these sources are fluctuated during stress conditions (Onywera et al., 2019).

**Figure 5:**
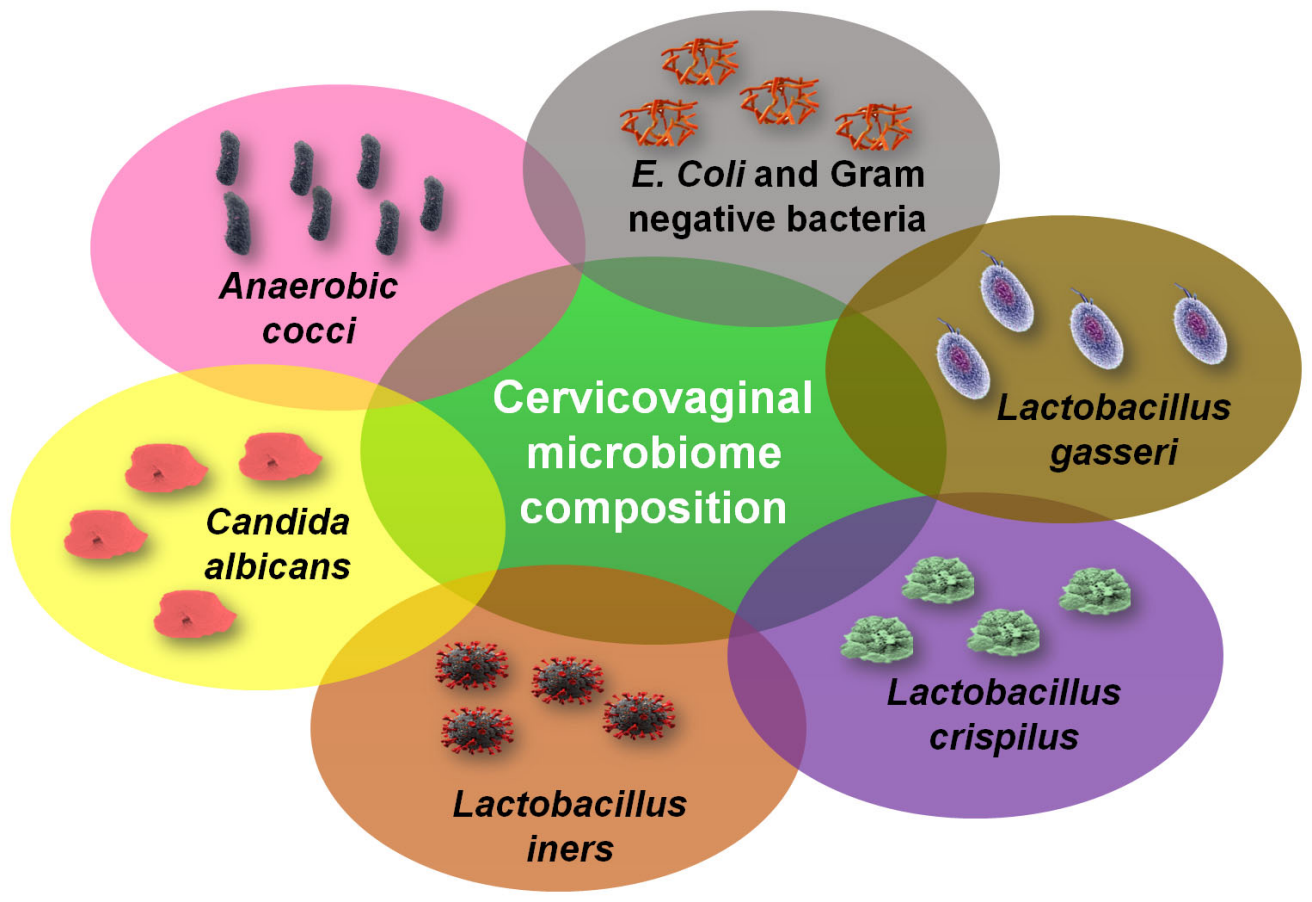
The cervicovaginal microbiome composition. The cervical fluid contains different types of microbiotas called as the cervicovaginal microbiome that mainly include *Anaerobic cocci*, *Candida albicans* and different species of *Lactobacillus*.

The cervicovaginal microbiome composition presented in **Figure 5** shows the different types of microbiotas engaged in the cervicovaginal microbiome that mainly include Anaerobic cocci, *Candida albicans* and different species of *Lactobacillus*. In general, the *Lactobacillus* spp. are most abundant in the cervicovaginal microbiota that causes acidic and antimicrobial activity for external viruses and pathogens (Audirac-Chalifour et al., 2016). Some species of *Lactobacillus* such as *L. gasseri*, *L. crispatus*, and *L. jensenii* have been known to produce lactic acid and hydrogen peroxide which acts to inhibit the growth of other bacteria, viruses, and external pathogens. The cervicovaginal microbiome profile and their composition is highly dynamic, and this composition changes due to the hormonal fiuctuations which take place during the reproductive cycle of women and are highly associated with stress conditions (Castanheira et al., 2021). This change also occurs due to the influence of physico-social and psychological activities like sexual activity, use of oral contraceptives, vaginal douching, disturbance in biological clock and late-night working schedules, and exhaustive travels. It is also influenced by pathophysiological conditions like oxidative stress, diabetes mellitus, stress anxieties, pregnancy, and lactation. Cervical cancer screening is often abnormal in pregnant women with an altered rate of cytological abnormalities which is reported to be similar to some extent among nonpregnant gynecological patients (Van Calsteren et al., 2005). During puberty, estrogen level raises to a notable level which promotes physiological growth, cells and tissues proliferation, maturation and differentiation, and accumulation of glycogen and precursor of glycogen in the vaginal epithelium region (Wu et al., 2021). Glycogen being the linear polymer of glucose is catabolized by the enzyme α-amylase which converts the linear chain of glycogen into a monomeric unit of glucose and amylopectin (branch degradation point) in the vaginal epithelium where the latter serves as carbon sources for lactic acid fermentation metabolism by *Lactobacillus* species (Lin et al., 2020). Lactic acid has two isomers (D and L forms) of them D-lactic acid is more protective in nature and acts against infections. Along with the production of lactic acid, *Lactobacillus* spp. also act to produce antimicrobial peptides such as bacteriocins and biosurfactants which have inhibitory effects on pathogen growth and thus help in establishing low pH in the cervicovaginal area. Whereas when pH increases it decreases the survival rate of the vaginal microbiome and reduces susceptibility towards viral infections (Andralojc et al., 2021). The dynamic balance of the cervicovaginal microbiome could affect the host physiological, hormonal, and immune system *via* affecting the estrogen levels especially during menstruation and reproductive state (Macintyre et al., 2015; Chen et al., 2021). Menopausal women lack of estrogen that causes an increase in anaerobic vaginal microflora and a decrease in *Lactobacillus* population, which affects the release of inflammatory cytokines, chemokines, and vaginal antimicrobial peptides (Torcia, 2019). Thus, estrogen is considered as an important physiological mediator which helps in the maturation of the vaginal epithelium cells to produce α-amylase. This enzyme acts to degrade glycogen into simple sugars such as malto-tetraose, malto-triose, and α-dextrins. Since *Lactobacillus* could not break down glycogen, it relies on glycogen degradation sugar products for growing and forming colonies in the vagina. This causes production of lactic acid in the vaginal epithelium and that inhibits the growth of other bacterial species (Spear et al., 2014). The production of lactic acid exerts protective effects by reducing the cytotoxicity of NK cells, inducing the synthesis of anti-inflammatory cytokine IL-10, and reducing the synthesis of dendritic cell pro-inflammatory cytokine IL-12 (Sun et al., 2017). *Lactobacillus* thus been established to play protective role in maintaining the physiological functions in the female reproductive tract and that the vaginal microbiota composition may influence localized immunity and clearance of the HPV and impart to the cervical carcinogenesis and progression (Audirac-Chalifour et al., 2016). The cervicovaginal microenvironment consisting of *Lactobacillus* maintains the dynamic balance of the cervix and vagina region microflora and that increased microbial diversity may cause the augmented inflammatory response through production of pro-inflammatory cytokines and chemokines (Norenhag et al., 2020). This situation promotes immune dysregulation in the cervicovaginal region and make the area a susceptible site for tumor development. This altered microbial growth and metabolic state could also promote the replication and transcription of HPV that may increase prospect of the developing cervical cancer. (Moscicki et al., 2020) investigated the variations in host immune response related to the HPV status and microbiome by analyzing the expression profiles of thirteen different inflammatory cytokines. The study showed that nine inflammatory cytokines (IL-4, IL-5, IL-10, IL-12, IL-13, IFN-γ, IFN-α2, MIP-1α, and TNF-α) were significantly increased in the HPV clearance state and that infection with *Gardnerella vaginalis* was associated with higher levels of inflammatory cytokines. The study also recommended that therapies such as probiotics or pro-inflammatory agents for treatment of persistent HPV may be additionally beneficial in treating the infection and reducing the risk of cervical cancer (Moscicki et al., 2020). Therefore, studies need to further establish the relationship between the growth and metabolic activities of bacterial populations in the cervicovaginal region and cervical cancer development. This review extended to establish the link between the cervicovaginal microbiota with physiological and hormonal distress and inflammation with the development and progression of cervical cancer in view of physiological stress especially stress insomnia.

## Stress insomnia, microbiome, and cervical cancer

Sleep is an active and necessary physiological process for life that plays fundamental role for physical, mental, and emotional health. Sleep necessity and its patterns are attributed to a complex interplay between age chronology, stage of maturation, behavioral, socio-environmental, and genetic factors (Garbarino et al., 2021). Sleep apnea and insomnia are resultant of the work cultures and societal concerns that contribute to chronic phenomenon including carcinogenic developments, especially observed in breast and ovarian cancer (Fiorentino et al., 2011; Clevenger et al., 2012; Büttner-Teleagă et al., 2021). A bacterial cell wall component muramyl peptide caused activation of the immune system and induced the release of sleep-regulatory cytokines as the principal regulators of the inflammation. Other microbial factors such as the endotoxin lipopolysaccharide, IL-1, TNF-α, prostaglandins, growth factors and growth hormone-releasing hormone act as critical mediators of inflammation (Zielinski and Krueger, 2011). The immune system may signal to the brain that interrelate to other sleep-regulatory factors such as neurotransmitters (dopamine, serotonin, acetylcholine, norepinephrine, and histamine), melatonin hormone, HPA axis, neuropeptides, and nucleosides (Besedovsky et al., 2019). Sleep is a self-organizing event for developing neuronal networks that acts like recharging these sleep-regulatory substances in response to cells exposed to acute to chronic pathological conditions, especially the interplay of IL-1, IL-1β and TNF-α (Taishi et al., 2008; Rockstrom et al., 2018).

Research have drawn considerable attention to the association between stress insomnia as the risk factor to induce cervical cancer and their effects on the cervicovaginal microbiome as well. Studies focused on the preventive and therapeutic manifestations for the prevention of infection and cervical cancer using probiotics (Kyrgiou et al., 2017; Gupta et al., 2019; Curty et al., 2020; Lin et al., 2020). Obstructed sleep and deprivation have been recognized as the high-risk factor for tumor response with a significant association between sleep deprivation and the risk for several cancers including breast, colorectal, and prostate cancer (Garbarino et al., 2021). World Health Organization (WHO) has accorded that probiotics are live microorganisms (found in the gut) that on being administered in valid quantities confer a health benefit on the host (Hong et al., 2020). Probiotic species including *Lactobacillus, Lactococcus, Enterococcus, Streptococcus*, and *Bifidobacterium* have shown beneficial effects with the *Lactobacillus* genus as the best-known intravaginal beneficial probiotic species (Fijan, 2014). In low immune response and stress conditions, the species of *Anaerobic cocci, Candida albicans, E. coli*, and Gram-negative *Lactobacillus, Bifidobacterium*, and *Streptococcus* are capable of shifting the composition of the host microbiome (Glaser et al., 1987; Ghosh and Pramanik, 2021). Probiotics containing species of lactobacilli have the capabilities to treat urogenital and the cervicovaginal infections to improve and augment the vaginal flora, the immune responses, and the inflammatory state in the host. The mechanism of action mainly involves acidification (lowered pH) of the vaginal region, prevention of bacterial persistence and adhesion, and synergistic action with the host immune system (Dietert, 2021). *Lactobacillus* spp. catalyze the decomposition of carbohydrates into lactic acid and CO_2_ that maintains an acidic intravaginal microflora thus preventing vaginal colonization of harmful microorganisms and preventing the growth of mainly Enterobacteria, *E. coli, Candida*, and *G. vaginalis* (Cadieux et al., 2002). Even though conclusive evidences are lacking to show the efficacy of probiotics yet they appear to be an alternative complementary treatment for bacterial vaginosis (BV) and complicated vulvovaginal candidiasis (VVC). Probiotics are known to help in other sexually transmitted diseases as well by virtue that they do not induce inflammation, control bacterial resistance, and have no adverse effects as such (Fijan, 2014). An imbalance between Gram-negative microbes (mainly *Candida* spp.*, G. vaginalis*) and Gram-positive microbes (mainly *Lactobacillus* spp.) in the vaginal microbiome has been associated with vaginal infections like BV and VVC (Putta et al., 2018). The impacts of probiotics on cytological changes of the uterine cervix and on HPV infection were reported in a study with fifty-one individuals infected with HPV and some other vaginal infections (Ditmer et al., 2021). A group of women daily received the different probiotics containing *L. paracasei* strain Shirota, and another group of women made up the control group. After 3 months, HPV and other vaginal infections were cleared in 25% of the women who were supplemented with probiotics while the clearance rate was 7.7% in the control group (without probiotics). Whereas after 6 months, the clearance rates increased to 29% in probiotic-supplemented women and 19% in the control group (Ditmer et al., 2021). Yet the HPV-infection and vaginal infection clearance rate was assumed to be not very efficient in the host and required a greater amount of the probiotics for the clearance of viruses and other infectious particles (Linn et al., 2019).

**Figure 6:**
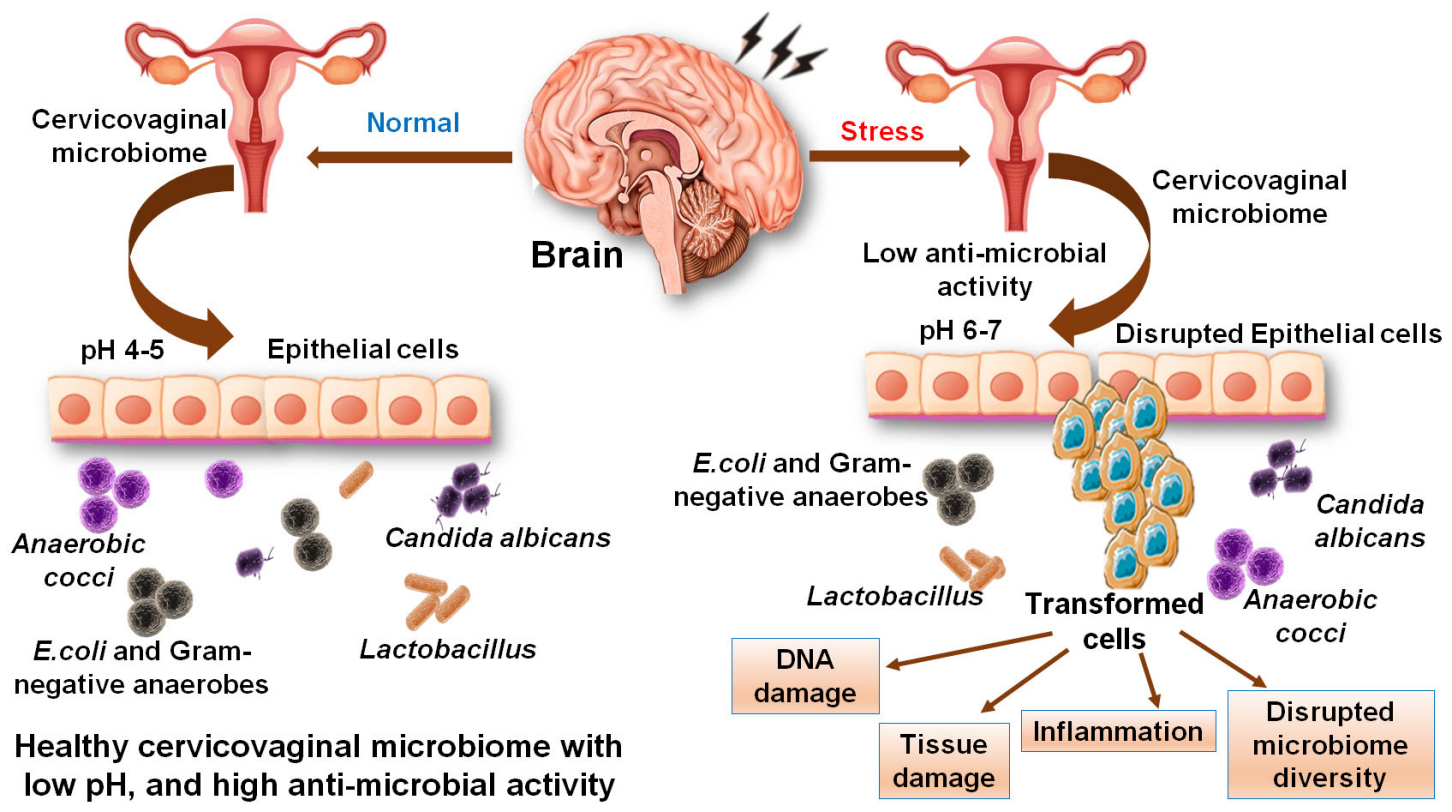
Effect of stress on cervical physiology and function and on the cervicovaginal microbiome. Stress induces alterations in the pH that causes disruption of cervical epithelium and induces migration of cells. Stress also causes alterations in the cervical microbiome and a reduction in healthy microflora.

Treatment of HeLa cells with supernatants of *L. rhamnosus*, *L. crispatus*, *C. albicans*, and other probiotics were shown to cause potent inhibitory effects on metastasis in the epithelial region of the cervix. It caused notable reductions in the expression of the CASP3 gene (apoptosis executioner caspase), MMP2 (degrade type 4 collagen), and MMP9 (that breakdown extracellular matrix protein) (Cha et al., 2012). *Bifidobacterium adolescentis* SPM1005-A was found to exert an antiviral activity in SiHa cervical cell line (that expressed HPV type 16) and showed preventive aspects in this cancer. Treating HeLa cells with this bacterium caused a notable reduction in the expression of E6 and E7 oncogenes (associated with the tumor suppressors protein and retinoblastoma protein respectively). The probiotic strains, *L. gasseri* 3396 and *L. cripatus* 2743, showed protective effects on supplementation to cells by suppressing the mRNA level of E6 and E7l. On a comparative account, *L. gasseri* 2743 showed potent effects as compared to *L. gasseri* 3396 that showed a smaller inhibitory impact on the E6 gene without any noteworthy consequences on the E7 gene. However, the effects of probiotics were observed to be lowered during stress and anxieties condition as compared to normal conditions (Hernández-Quiroz et al., 2021). Poor sleep quality was also present in cervical cancer patients with an almost twice as many women with cervical cancer had poor sleep quality (Tian et al., 2015). The mechanism behind the development of sleep disorders was associated with the release of cytokines like TNFα, IL-6 and C-reactive protein (CRP). Cervical cancers patents under radiotherapy showed poor sleep quality with effects on daytime dysfunction, sleep latency, and sleep quality (İlhan et al., 2017). Female patients with cancers of ovary, breast and cervix were reported with more severe insomnia and problems with daily life. The ratio of stress response and anxiety symptoms were variable in different cancers, yet it remained more distressing among breast cancer patients (Ho et al., 2021). Thus, reducing the stress and applying mindfulness practices showed beneficial effects on patients with insomnia and that was a considered as a supplementary treatment to cervical cancer (Zhang et al., 2019). Preceding discussions conclude that stress and insomnia play critical role in managing the healthy microbiota that has significant impacts on improving the environment of the cervicovaginal microbiome and preventing women from cancer promotion and cervical lesions. A prospective summary of the effect of stress on cervical physiology and function and the role of the cervicovaginal microbiome is presented in **Figure 6**. It concluded that stress induced pH alterations may causes disruption of cervical epithelium and cancerous migration. This review attempted to identify the interconnected links between the stress and insomnia with the cervicovaginal microbiome and cervical cancer. It is presented that the cervicovaginal microbiota profiles helps in managing the cervicovaginal health through the protective effects of microbial species like *Lactobacillus*. Stressors and insomnia are highly likely to be associated with the cervicovaginal microbiota disturbances that could cause inflammation and exaggerated response of HPV towards carcinogenic developments in the cervix.

## Conclusions and future direction

We conducted a systematic review on the vaginal microbiome in correlation with cervical cancer and stress. Studies revealed the relationship between the risk of cervix lesions and cervical cancer promotion with physiological and psychological stress and insomnia. Their interplay is considered to be associated with an increased risk of cancer-specific mortality in cervical cancer patients. Patients with prospects of developing depression and physiological and psychological stress are prone to develop chronic disease conditions which worsen in conditions with comorbidities like oxidative and nitrosative stress, ischemic heart disease, cerebrovascular disease, and diabetes mellitus. Thus, the question of stress and insomnia becomes even more important as a lifestyle and behavioral factor that has greatly imparted to work culture and society. These factors have modulated the expression and inflammatory signaling pathways in cells that exaggerated the cellular growth and signaling systems leading to aggressive and metastatic cervical cancer. It is noteworthy that the literature reported in the interconnection between the cervicovaginal microbiome, stress and cervical cancer are largely reported in the past decade that indicates that changing lifestyle and work cultures have significantly affected the course of metabolism and physiology and pathophysiology.

Further studies are vital to examine how the cervicovaginal microbiome influences the immune response and therapeutic responses in stress insomnia-related gynecologic cervical cancers. It also needs to elaborate whether the management of microbiota could enhance the response to therapy in preclinical models and in clinical settings. Recent developments in microbiome sequencing technologies and bioinformatic tools may fuel these advanced studies for understanding the human microbiome and analysing the different species of bacteria and clusters imparting in cervical cancer. Identification of bacteria into operational taxonomic units and identifying the variations between the microbes inside the vulva region would be necessitated which may also involve whole-genome sequencing of microbial DNA from normal and patient samples. A comparative analysis would be more appropriate to establish the correlation between factors affecting long-term stress in working women and the high risk of cervical cancer. Other aspects of advance studies involving genomics and proteomics of genes and proteins and their structure and function in the vaginal microbiome would be able to unfold the molecular mechanisms. In normal and healthy conditions, the vaginal microbiome secretes functional protein and exhibits a strong relationship with immune response and showed an ability to effectively respond against viruses and pathogens. But in stressful conditions, it secretes much more types of inflammatory cytokines and chemokines, and free radicals through the endoplasmic reticulum of epithelial cells of the cervicovaginal tissues. This causes protein misfolding which stands responsible for improper function and further affects the cervicovaginal microbiome density as well. Thus, genomics and proteomics applications may be utilized to analyze and understand the role of the microbiota on the microbial species proliferation and growth and their metabolic activities in the cervical region. Summarily, this comprehensive review emphasizes the characteristics of stress and inflammatory responses in the cervicovaginal tissues under stress conditions which lead to chronicity and cancer, as well as suggests the potential targets for preventive management of stress, inflammation, and cancer progression.

## Author contributions

RR compiled the literature and performed review work. MN performed the secondary analysis. DJ and NG assisted in literature analysis and manuscript preparation. SKM overall managed to organize the work, finalized the manuscript, and managed the manuscript submission. All authors contributed to the article and approved the submitted version.

## References

[B1] Anahtar M. N. Byrne E. H. Doherty K. E. Bowman B. A. Yamamoto H. S. Soumillon M.. (2015). Cervicovaginal bacteria are a major modulator of host inflammatory responses in the female genital tract. Immunity 42, 965–976. doi: 10.1016/j.immuni.2015.04.019 25992865 PMC4461369

[B2] Andralojc K. M. Molina M. A. Qiu M. Spruijtenburg B. Rasing M. Pater B.. (2021). Novel high-resolution targeted sequencing of the cervicovaginal microbiome. BMC Biol. 19, 1–18. doi: 10.1186/s12915-021-01204-z 34915863 PMC8680041

[B3] Ash H. Smith T. E. Buchanan-Smith H. M. (2021). The long-term impact of infant rearing background on the behavioural and physiological stress response of adult common marmosets (*Callithrix jacchus*). Appl. Anim. Behav. Sci. 234, 105169. doi: 10.1016/j.applanim.2020.105169 PMC4727505 26912940

[B4] Audirac-Chalifour A. Torres-Poveda K. Bahena-Román M. Téllez-Sosa J. Martínez-Barnetche J. Cortina-Ceballos B.. (2016). Cervical microbiome and cytokine profile at various stages of cervical cancer: a pilot study. PloS One 11, e0153274. doi: 10.1371/journal.pone.0153274 27115350 PMC4846060

[B5] Beharee N. Shi Z. Wu D. Wang J. (2019). Diagnosis and treatment of cervical cancer in pregnant women. Cancer Med. 8, 5425–5430. doi: 10.1002/cam4.2435 31385452 PMC6745864

[B6] Besedovsky L. Lange T. Haack M. (2019). The sleep-immune crosstalk in health and disease. Physiol. Rev. 99, 1325–1380. doi: 10.1152/physrev.00010.2018 30920354 PMC6689741

[B7] Burd E. M. (2003). Human papillomavirus and cervical cancer. Clin. Microbiol. Rev. 16, 1–17. doi: 10.1128/CMR.16.1.1-17.2003 12525422 PMC145302

[B8] Büttner-Teleagă A. Kim Y. T. Osel T. Richter K. (2021). Sleep disorders in cancer-a systematic review. Int. J. Environ. Res. Public Health 18, 1–38. doi: 10.3390/ijerph182111696. PMC8583058 34770209

[B9] Cadieux P. Burton J. Gardiner G. Braunstein I. Bruce A. W. Kang C. Y.. (2002). Lactobacillus strains and vaginal ecology. Jama 287, 1940–1941. doi: 10.1001/jama.287.15.1940 11960535

[B10] Carrero Y. N. Callejas D. E. Mosquera J. A. (2021). *In situ* immunopathological events in human cervical intraepithelial neoplasia and cervical cancer. Trans. Oncol 14, 101058. doi: 10.1016/j.tranon.2021.101058 PMC7937982 33677234

[B11] Castanheira C. P. Sallas M. L. Nunes R. A. L. Lorenzi N. P. C. Termini L. (2021). Microbiome and cervical cancer. Pathobiology 88, 187–197. doi: 10.1159/000511477 33227782

[B12] Cha M.-K. Lee D. An H.-M. Lee S.-W. Shin S.-H. Kwon J.-H.. (2012). Antiviral activity of bifidobacterium adolescentis SPM1005-a on human papillomavirus typeo 16. BMC Med. 10, 72. doi: 10.1186/1741-7015-10-72 22788922 PMC3409845

[B13] Chee W. J. Y. Chew S. Y. Than L. T. L. (2020). Vaginal microbiota and the potential of lactobacillus derivatives in maintaining vaginal health. Microb. Cell Fact 19, 203. doi: 10.1186/s12934-020-01464-4 33160356 PMC7648308

[B14] Chen Y. Hong Z. Wang W. Gu L. Gao H. Qiu L.. (2019). Association between the vaginal microbiome and high-risk human papillomavirus infection in pregnant Chinese women. BMC Infect. Dis. 19, 677. doi: 10.1186/s12879-019-4279-6 31370796 PMC6669982

[B15] Chen G. Qiu L. Gao J. Wang J. Dang J. Li L.. (2021). Stress hormones: Emerging targets in gynecological cancers. Front. Cell Dev. Biol. 9. doi: 10.3389/fcell.2021.699487 PMC8299464 34307378

[B16] Chen H. Zhang W. Cheng X. Guo L. Xie S. Ma Y.. (2017). β2-AR activation induces chemoresistance by modulating p53 acetylation through upregulating Sirt1 in cervical cancer cells. Cancer Sci. 108, 1310–1317. doi: 10.1111/cas.13275 28498637 PMC5497720

[B17] Chorna N. Romaguera J. Godoy-Vitorino F. (2020). Cervicovaginal microbiome and urine metabolome paired analysis reveals niche partitioning of the microbiota in patients with human papilloma virus infections. Metabolites 10, 36. doi: 10.3390/metabo10010036 31952112 PMC7022855

[B18] Clevenger L. Schrepf A. Christensen D. Degeest K. Bender D. Ahmed A.. (2012). Sleep disturbance, cytokines, and fatigue in women with ovarian cancer. Brain Behav. Immun. 26, 1037–1044. doi: 10.1016/j.bbi.2012.04.003 22543257 PMC3434312

[B19] Cohen M. Karrington B. Trachtman H. Salas-Humara C. (2021). Allostatic stress and inflammatory biomarkers in transgender and gender expansive youth: Protocol for a pilot cohort study. JMIR Res. Protoc. 10, e24100. doi: 10.2196/24100 34009131 PMC8173394

[B20] Curty G. De Carvalho P. S. Soares M. A. (2020). The role of the cervicovaginal microbiome on the genesis and as a biomarker of premalignant cervical intraepithelial neoplasia and invasive cervical cancer. Int. J. Mol. Sci. 21, 222. doi: 10.3390/ijms21010222 PMC6981542 31905652

[B21] Cvitanović H. Milošević M. Bukvić-Bešlić I. Lugović-Mihić L. (2020). Determination of psychological stress, serum immune parameters, and cortisol levels in patients with human papilloma virus. Clin. Ther. 42, 783–799. doi: 10.1016/j.clinthera.2020.03.017 32340917

[B22] Dietert R. R. (2021). Microbiome first medicine in health and safety. Biomedicines 9, 1–25. doi: 10.3390/biomedicines9091099 PMC8468398 34572284

[B23] Ditmer M. Gabryelska A. Turkiewicz S. Białasiewicz P. Małecka-Wojciesko E. Sochal M. (2021). Sleep problems in chronic inflammatory diseases: Prevalence, treatment, and new perspectives. J. Clin. Med. 2022, 11–67. doi: 10.3390/jcm11010067 PMC8745687 35011807

[B24] Eimonte M. Eimantas N. Daniuseviciute L. Paulauskas H. Vitkauskiene A. Dauksaite G.. (2021). Recovering body temperature from acute cold stress is associated with delayed proinflammatory cytokine production *in vivo* . Cytokine 143, 155510. doi: 10.1016/j.cyto.2021.155510 33820701

[B25] Elemam Y. Bakhit I. (2018). Screening for high risk human papilloma virus (HR-HPV), cytomegalovirus, herpes simplex virus (HS-2) and Epstein-Barr virus among Sudanese females with cervical cancer. J. Exp. Zool India 21, 571–576. Available at: http://hdl.handle.net/123456789/431.

[B26] Everly G. S. Lating J. M. (2019). “The anatomy and physiology of the human stress response,” in A clinical guide to the treatment of the human stress response (Springer New York, NY:Springer), 19–56. doi: 10.1007/978-1-4614-5538-7

[B27] Ferrandina G. Mantegna G. Petrillo M. Fuoco G. Venditti L. Terzano S.. (2012). Quality of life and emotional distress in early stage and locally advanced cervical cancer patients: a prospective, longitudinal study. Gynecol Oncol. 124, 389–394. doi: 10.1016/j.ygyno.2011.09.041 22035809

[B28] Fijan S. (2014). Microorganisms with claimed probiotic properties: an overview of recent literature. Int. J. Environ. Res. Public Health 11, 4745–4767. doi: 10.3390/ijerph110504745 24859749 PMC4053917

[B29] Fiorentino L. Rissling M. Liu L. Ancoli-Israel S. (2011). The symptom cluster of sleep, fatigue and depressive symptoms in breast cancer patients: Severity of the problem and treatment options. Drug Discovery Today Dis. Models 8, 167–173. doi: 10.1016/j.ddmod.2011.05.001 PMC3228259 22140397

[B30] Garbarino S. Lanteri P. Bragazzi N. L. Magnavita N. Scoditti E. (2021). Role of sleep deprivation in immune-related disease risk and outcomes. Commun. Biol. 4, 1–17. doi: 10.1038/s42003-021-02825-4 PMC8602722 34795404

[B31] Ghosh S. Pramanik S. (2021). Structural diversity, functional aspects and future therapeutic applications of human gut microbiome. Arch. Microbiol. 203, 5281–5308. doi: 10.1007/s00203-021-02516-y 34405262 PMC8370661

[B32] Glaser R. Rice J. Sheridan J. Fertel R. Stout J. Speicher C.. (1987). Stress-related immune suppression: Health implications. Brain Behav. Immun. 1, 7–20. doi: 10.1016/0889-1591(87)90002-X 2837297

[B33] Gupta S. Kakkar V. Bhushan I. (2019). Crosstalk between vaginal microbiome and female health: a review. Microbial Pathogenesis 136, 103696. doi: 10.1016/j.micpath.2019.103696 31449855

[B34] Hernández-Quiroz F. Murugesan S. Velazquez-Martínez C. Villalobos-Flores L. E. Maya-Lucas O. Piña-Escobedo A.. (2021). The vaginal and fecal microbiota of a murine cervical carcinoma model under synergistic effect of 17β-estradiol and E7 oncogene expression. Microbial Pathogenesis 152, 104763. doi: 10.1016/j.micpath.2021.104763 33529736

[B35] Ho D. Kim S. Y. Kim S. I. Kim S. Y. Lim W. J. (2021). Insomnia, anxiety, and depression in patients first diagnosed with female cancer. Psychiatry Investig. 18, 755–762. doi: 10.30773/pi.2021.0090 PMC8390945 34380297

[B36] Hong J. Chen J. Kan J. Liu M. Yang D. (2020). Effects of acupuncture treatment in reducing sleep disorder and gut microbiota alterations in PCPA-induced insomnia mice. Evidence-Based Complementary Altern. Med. 2020, 1–14. doi: 10.1155/2020/3626120 PMC7647758 33178314

[B37] Huang T. Tworoger S. S. Hecht J. L. Rice M. S. Sood A. K. Kubzansky L. D.. (2016). Association of ovarian tumor β2-adrenergic receptor status with ovarian cancer risk factors and survival. Cancer Epidemiol. Biomarkers Prev. 25, 1587–1594. doi: 10.1158/1055-9965.EPI-16-0534 27587791 PMC5135562

[B38] Huzard D. Rappeneau V. Meijer O. C. Touma C. Arango-Lievano M. Garabedian M. J.. (2021). Experience and activity-dependent control of glucocorticoid receptors during the stress response in large-scale brain networks. Stress 24, 130–153. doi: 10.1080/10253890.2020.1806226 32755268 PMC7907260

[B39] İlhan T. T. Uçar M. G. Gül A. İlhan T. S. Yavaş G. Çelik Ç. (2017). Sleep quality of endometrial cancer survivors and the effect of treatments. Turkish J. Obstetrics Gynecol 14, 243–248. doi: 10.4274/tjod.59265 PMC5780569 29379668

[B40] Ishfaq P. M. Mishra A. Mishra S. Ahmad Z. Gayen S. Jain S. K.. (2021). Inonotus obliquus aqueous extract suppresses carbon tetrachloride-induced hepatic injury through modulation of antioxidant enzyme system and anti-inflammatory mechanism. Clin. Cancer Drugs 8, 122–136. doi: 10.2174/2212697X08666211130130119

[B41] Ishfaq P. M. Mishra S. Mishra A. Ahmad Z. Gayen S. Jain S. K.. (2022). Inonotus obliquus aqueous extract prevents histopathological alterations in liver induced by environmental toxicant microcystin. Curr. Res. Pharmacol. Drug Discovery 3, 100118. doi: 10.1016/j.crphar.2022.100118 PMC9389225 35992377

[B42] Karvonen H. Arjama M. Kaleva L. Niininen W. Barker H. Koivisto-Korander R.. (2020). Glucocorticoids induce differentiation and chemoresistance in ovarian cancer by promoting ROR1-mediated stemness. Cell Death Dis. 11, 1–12. doi: 10.1038/s41419-020-03009-4 32989221 PMC7522257

[B43] Kennedy B. Valdimarsdóttir U. Sundström K. Sparén P. Lambe M. Fall K.. (2014). Loss of a parent and the risk of cancer in early life: a nationwide cohort study. Cancer Causes Control 25, 499–506. doi: 10.1007/s10552-014-0352-z 24500176

[B44] Kim M. K. Lee B. Mishra S. K. Lee J. K. (2012). Abstract 653: Passive smoking facilitate the risk of CIN1 in high risk HPV-positive women: Korean HPV cohort study. Cancer Res. 72, 653–653. doi: 10.1158/1538-7445.AM2012-653

[B45] Kohli N. Dalal A. K. (2020). “Psychological recovery of women with cervical cancer: The role of cultural beliefs,” in Merging past, present, and future in cross-cultural psychology, (London:Garland Science) 348–364. doi: 10.1201/9781003077473

[B46] Kotas M. E. Medzhitov R. (2015). Homeostasis, inflammation, and disease susceptibility. Cell 160, 816–827. doi: 10.1016/j.cell.2015.02.010 25723161 PMC4369762

[B47] Kyrgiou M. Mitra A. Moscicki A.-B. (2017). Does the vaginal microbiota play a role in the development of cervical cancer? Trans. Res. 179, 168–182. doi: 10.1016/j.trsl.2016.07.004 PMC5164950 27477083

[B48] Lin D. Kouzy R. Abi Jaoude J. Noticewala S. S. Delgado Medrano A. Y. Klopp A. H.. (2020). Microbiome factors in HPV-driven carcinogenesis and cancers. PloS Pathog. 16, e1008524. doi: 10.1371/journal.ppat.1008524 32497113 PMC7271998

[B49] Linn Y. H. Thu K. K. Win N. H. H. (2019). Effect of probiotics for the prevention of acute radiation-induced diarrhoea among cervical cancer patients: a randomized double-blind placebo-controlled study. Probiotics Antimicrobial Proteins 11, 638–647. doi: 10.1007/s12602-018-9408-9 29550911

[B50] Lu D. Andrae B. Valdimarsdóttir U. Sundström K. Fall K. Sparén P.. (2019). Psychologic distress is associated with cancer-specific mortality among patients with cervical cancer. Cancer Res. 79, 3965–3972. doi: 10.1158/0008-5472.CAN-19-0116 31253667

[B51] Lu W. He F. Lin Z. Liu S. Tang L. Huang Y.. (2021). Dysbiosis of the endometrial microbiota and its association with inflammatory cytokines in endometrial cancer. Int. J. Cancer 148, 1708–1716. doi: 10.1002/ijc.33428 33285000

[B52] Lukac A. Sulovic N. Smiljic S. Ilic A. N. Saban O. (2018). The prevalence of the most important risk factors associated with cervical cancer. Mater Sociomed 30, 131–135. doi: 10.5455/msm.2018.30.131-135 PMC6029895 30061804

[B53] Macintyre D. A. Chandiramani M. Lee Y. S. Kindinger L. Smith A. Angelopoulos N.. (2015). The vaginal microbiome during pregnancy and the postpartum period in a European population. Sci. Rep. 5, 1–9. doi: 10.1038/srep08988 PMC4355684 25758319

[B54] Mcbride E. Tatar O. Rosberger Z. Rockliffe L. Marlow L. A. Moss-Morris R.. (2021). Emotional response to testing positive for human papillomavirus at cervical cancer screening: a mixed method systematic review with meta-analysis. Health Psychol. Rev. 15, 395–429. doi: 10.1080/17437199.2020.1762106 32449477

[B55] Mitra A. Macintyre D. A. Marchesi J. R. Lee Y. S. Bennett P. R. Kyrgiou M. (2016). The vaginal microbiota, human papillomavirus infection and cervical intraepithelial neoplasia: what do we know and where are we going next? Microbiome 4, 1–15. doi: 10.1186/s40168-016-0203-0 27802830 PMC5088670

[B56] Moreno-Smith M. Lee S. J. Lu C. Nagaraja A. S. He G. Rupaimoole R.. (2013). Biologic effects of dopamine on tumor vasculature in ovarian carcinoma. Neoplasia 15, 502–510. doi: 10.1593/neo.121412 23633922 PMC3638353

[B57] Morey J. N. Boggero I. A. Scott A. B. Segerstrom S. C. (2015). Current directions in stress and human immune function. Curr. Opin. Psychol. 5, 13–17. doi: 10.1016/j.copsyc.2015.03.007 26086030 PMC4465119

[B58] Moscicki A.-B. Shi B. Huang H. Barnard E. Li H. (2020). Cervical-vaginal microbiome and associated cytokine profiles in a prospective study of HPV 16 acquisition, persistence, and clearance. Front. Cell. Infection Microbiol. 10, 528. doi: 10.3389/fcimb.2020.569022 PMC7546785 33102255

[B59] Myint K. Jacobs K. Myint A. M. Lam S. K. Henden L. Hoe S. Z.. (2021). Effects of stress associated with academic examination on the kynurenine pathway profile in healthy students. PloS One 16, e0252668. doi: 10.1371/journal.pone.0252668 34081742 PMC8174692

[B60] Nelson E. L. Wenzel L. B. Osann K. Dogan-Ates A. Chantana N. Reina-Patton A.. (2008). Stress, immunity, and cervical cancer: biobehavioral outcomes of a randomized clinical trial [corrected]. Clin. Cancer Res. 14, 2111–2118. doi: 10.1158/1078-0432.CCR-07-1632 18381952 PMC4572837

[B61] Nie H. Bu F. Xu J. Li T. Huang J. (2020). 29 immune-related genes pairs signature predict the prognosis of cervical cancer patients. Sci. Rep. 10, 1–16. doi: 10.1038/s41598-020-70500-5 32843657 PMC7447790

[B62] Nieves-Ramírez M. Partida-Rodríguez O. Moran P. Serrano-Vázquez A. Pérez-Juárez H. Pérez-Rodríguez M.. (2021). Cervical squamous intraepithelial lesions are associated with differences in the vaginal microbiota of Mexican women. Microbiol. Spectr. 9, e00143–e00121. doi: 10.1128/Spectrum.00143-21 34643408 PMC8515943

[B63] Norenhag J. Du J. Olovsson M. Verstraelen H. Engstrand L. Brusselaers N. (2020). The vaginal microbiota, human papillomavirus and cervical dysplasia: a systematic review and network meta-analysis. BJOG: Int. J. Obstetrics Gynaecol 127, 171–180. doi: 10.1111/1471-0528.15854 31237400

[B64] Onywera H. Williamson A.-L. Mbulawa Z. Z. Coetzee D. Meiring T. (2019). Factors associated with the composition and diversity of the cervical microbiota of reproductive-age black south African women: A retrospective cross-sectional study. PeerJ 7, e7488. doi: 10.7717/peerj.7488 31435492 PMC6698374

[B65] Otter S. Chatterjee J. Stewart A. Michael A. (2019). The role of biomarkers for the prediction of response to checkpoint immunotherapy and the rationale for the use of checkpoint immunotherapy in cervical cancer. Clin. Oncol. 31, 834–843. doi: 10.1016/j.clon.2019.07.003 31331818

[B66] Paz M. Gomes A. L. J. Islam M. T. Tabrez S. Jabir N. R. Alam M. Z.. (2018). Assessment of chemotherapy on various biochemical markers in breast cancer patients. J. Cell Biochem. 119, 2923–2928. doi: 10.1002/jcb.26487 29120088

[B67] Paz M. Gomes Júnior A. L. De Alencar M. Tabrez S. Islam M. T. Jabir N. R.. (2019). Effect of diets, familial history, and alternative therapies on genomic instability of breast cancer patients. Appl. Biochem. Biotechnol. 188, 282–296. doi: 10.1007/s12010-018-2918-9 30430345

[B68] Putta S. Yarla N. S. Lakkappa D. B. Imandi S. B. Malla R. R. Chaitanya A. K.. (2018). “Probiotics: Supplements, food, pharmaceutical industry,” in Therapeutic, probiotic, and unconventional foods (Academic Press, USA: Elsevier), 15–25. doi: 10.1016/B978-0-12-814625-5.00002-9

[B69] Ravel J. Gajer P. Abdo Z. Schneider G. M. Koenig S. S. Mcculle S. L.. (2011). Vaginal microbiome of reproductive-age women. Proc. Natl. Acad. Sci. U.S.A. 108 Suppl 1, 4680–4687. doi: 10.1073/pnas.1002611107 20534435 PMC3063603

[B70] Reiche E. M. Nunes S. O. Morimoto H. K. (2004). Stress, depression, the immune system, and cancer. Lancet Oncol. 5, 617–625. doi: 10.1016/S1470-2045(04)01597-9 15465465

[B71] Rockstrom M. D. Chen L. Taishi P. Nguyen J. T. Gibbons C. M. Veasey S. C.. (2018). Tumor necrosis factor alpha in sleep regulation. Sleep Med. Rev. 40, 69–78. doi: 10.1016/j.smrv.2017.10.005 29153862 PMC5955790

[B72] Russell G. Lightman S. (2019). The human stress response. Nat. Rev. Endocrinol. 15, 525–534. doi: 10.1038/s41574-019-0228-0 31249398

[B73] Segerstrom S. C. Miller G. E. (2004). Psychological stress and the human immune system: a meta-analytic study of 30 years of inquiry. Psychol. Bull. 130, 601–630. doi: 10.1037/0033-2909.130.4.601 15250815 PMC1361287

[B74] Shannon B. Yi T. Perusini S. Gajer P. Ma B. Humphrys M.. (2017). Association of HPV infection and clearance with cervicovaginal immunology and the vaginal microbiota. Mucosal Immunol. 10, 1310–1319. doi: 10.1038/mi.2016.129 28120845 PMC5526752

[B75] Shi M. Du L. Liu D. Qian L. Hu M. Yu M.. (2012). Glucocorticoid regulation of a novel HPV-E6-p53-miR-145 pathway modulates invasion and therapy resistance of cervical cancer cells. J. Pathol. 228, 148–157. doi: 10.1002/path.3997 22287315

[B76] Showalter A. Limaye A. Oyer J. L. Igarashi R. Kittipatarin C. Copik A. J.. (2017). Cytokines in immunogenic cell death: Applications for cancer immunotherapy. Cytokine 97, 123–132. doi: 10.1016/j.cyto.2017.05.024 28648866 PMC5572581

[B77] Sims T. T. Colbert L. E. Klopp A. H. (2021). The role of the cervicovaginal and gut microbiome in cervical intraepithelial neoplasia and cervical cancer. J. Immunother Precis. Oncol. 4, 72–78. doi: 10.36401/JIPO-20-17 35663536 PMC9153260

[B78] Spear G. T. French A. L. Gilbert D. Zariffard M. R. Mirmonsef P. Sullivan T. H.. (2014). Human α-amylase present in lower-genital-tract mucosal fluid processes glycogen to support vaginal colonization by lactobacillus. J. Infect. Dis. 210, 1019–1028. doi: 10.1093/infdis/jiu231 24737800 PMC4168305

[B79] Sung H. Ferlay J. Siegel R. L. Laversanne M. Soerjomataram I. Jemal A.. (2021). Global cancer statistics 2020: GLOBOCAN estimates of incidence and mortality worldwide for 36 cancers in 185 countries. CA: A Cancer J. Clin. 71, 209–249. doi: 10.3322/caac.21660 33538338

[B80] Sun S. Li H. Chen J. Qian Q. (2017). Lactic acid: no longer an inert and end-product of glycolysis. Physiology 32, 453–463. doi: 10.1152/physiol.00016.2017 29021365

[B81] Taishi P. Churchill L. De A. Obal F. Jr. Krueger J. M. (2008). Cytokine mRNA induction by interleukin-1beta or tumor necrosis factor alpha *in vitro* and *in vivo* . Brain Res. 1226, 89–98. doi: 10.1016/j.brainres.2008.05.067 18620339 PMC2642478

[B82] Tian J. Chen G. L. Zhang H. R. (2015). Sleep status of cervical cancer patients and predictors of poor sleep quality during adjuvant therapy. Supportive Care Cancer 23, 1401–1408. doi: 10.1007/s00520-014-2493-8 25370891

[B83] Torcia M. G. (2019). Interplay among vaginal microbiome, immune response and sexually transmitted viral infections. Int. J. Mol. Sci. 20, 266. doi: 10.3390/ijms20020266 30641869 PMC6359169

[B84] Van Calsteren K. Vergote I. Amant F. (2005). Cervical neoplasia during pregnancy: diagnosis, management and prognosis. Best Pract. Res. Clin. Obstet Gynaecol 19, 611–630. doi: 10.1016/j.bpobgyn.2005.03.002 15886059

[B85] Wang H. Jiang Y. Liang Y. Wei L. Zhang W. Li L. (2022). Observation of the cervical microbiome in the progression of cervical intraepithelial neoplasia. BMC Cancer 22, 362. doi: 10.1186/s12885-022-09452-0 35379200 PMC8981842

[B86] Wu S. Ding X. Kong Y. Acharya S. Wu H. Huang C.. (2021). The feature of cervical microbiota associated with the progression of cervical cancer among reproductive females. Gynecol Oncol. 163, 348–357. doi: 10.1016/j.ygyno.2021.08.016 34503848

[B87] Xiao X. Wu Z.-C. Chou K.-C. (2011). A multi-label classifier for predicting the subcellular localization of gram-negative bacterial proteins with both single and multiple sites. PloS One 6, e20592. doi: 10.1371/journal.pone.0020592 21698097 PMC3117797

[B88] Xu Y. Qiu Y. Yuan S. Wang H. (2020). Prognostic implication of human papillomavirus types in cervical cancer patients: a systematic review and meta-analysis. Infect. Agent Cancer 15, 66. doi: 10.1186/s13027-020-00332-5 33292343 PMC7648311

[B89] Yang Y. L. Liu L. Wang X. X. Wang Y. Wang L. (2014). Prevalence and associated positive psychological variables of depression and anxiety among Chinese cervical cancer patients: a cross-sectional study. PloS One 9, e94804. doi: 10.1371/journal.pone.0094804 24722558 PMC3983270

[B90] Yang Y. L. Liu L. Wang Y. Wu H. Yang X. S. Wang J. N.. (2013). The prevalence of depression and anxiety among Chinese adults with cancer: a systematic review and meta-analysis. BMC Cancer 13, 393. doi: 10.1186/1471-2407-13-393 23967823 PMC3765872

[B91] Yang D. Zhang J. Cui X. Ma J. Wang C. Piao H. (2022). Risk factors associated with human papillomavirus infection, cervical cancer, and precancerous lesions in Large-scale population screening. Front. Microbiol. 13. doi: 10.3389/fmicb.2022.914516 PMC9282163 35847094

[B92] Yeh Y.-C. (2021). Symptom distress, stress, and quality of life in the first year of gynaecological cancers: A longitudinal study of women in Taiwan. Eur. J. Oncol. Nurs. 53, 1–10. doi: 10.1016/j.ejon.2021.101984 34275743

[B93] Zeng X. T. Xiong P. A. Wang F. Li C. Y. Yao J. Guo Y. (2012). Passive smoking and cervical cancer risk: a meta-analysis based on 3,230 cases and 2,982 controls. Asian Pac J. Cancer Prev. 13, 2687–2693. doi: 10.7314/APJCP.2012.13.6.2687 22938442

[B94] Zhang H. Li Y. Li M. Chen X. (2019). A randomized controlled trial of mindfulness-based stress reduction for insomnia secondary to cervical cancer: Sleep effects. Appl. Nurs. Res. 48, 52–57. doi: 10.1016/j.apnr.2019.05.016 31266608

[B95] Zhang L. Pan J. Chen W. Jiang J. Huang J. (2020). Chronic stress-induced immune dysregulation in cancer: implications for initiation, progression, metastasis, and treatment. Am. J. Cancer Res. 10, 1294–1307. doi: ajcr0111320 PMC7269780 32509380

[B96] Zhou Z.-W. Long H.-Z. Cheng Y. Luo H.-Y. Wen D.-D. Gao L.-C. (2021). From microbiome to inflammation: The key drivers of cervical cancer. Front. Microbiol. 12. doi: 10.3389/fmicb.2021.767931 PMC8634716 34867901

[B97] Zielinski M. R. Krueger J. M. (2011). Sleep and innate immunity. Front. biosci (Scholar edition) 3, 632–642. doi: 10.2741/s176 PMC3645929 21196401

